# STAT5 inhibition induces TRAIL/DR4 dependent apoptosis in peripheral T-cell lymphoma

**DOI:** 10.18632/oncotarget.24698

**Published:** 2018-03-30

**Authors:** Haley M. Simpson, Aki Furusawa, Kavitha Sadashivaiah, Curt I. Civin, Arnob Banerjee

**Affiliations:** ^1^ Department of Medicine, University of Maryland School of Medicine, Baltimore, MD, USA; ^2^ Program in Oncology, Greenebaum Comprehensive Cancer Center, University of Maryland School of Medicine, Baltimore, MD, USA; ^3^ Center for Stem Cell Biology & Regenerative Medicine, University of Maryland School of Medicine, Baltimore, MD, USA; ^4^ Department of Physiology and Pediatrics, University of Maryland School of Medicine, Baltimore, MD, USA

**Keywords:** STAT5, JAK/STAT, pimozide, peripheral T cell lymphoma, apoptosis

## Abstract

Peripheral T-cell lymphoma (PTCL) is a rare, aggressive, heterogeneous, Non-Hodgkin's lymphoma with poor prognosis and inadequate response to current therapies. Recent sequencing studies indicate a prevalence of activating mutations in the JAK/STAT signaling pathway. Oncogenic mutations in STAT5B, observed in approximately one third of cases of multiple different PTCL subtypes, correlate with inferior patient outcomes. Therefore, interest in the development of therapeutic strategies for targeting STAT5 in PTCL is warranted. In this study, we show that the drug pimozide inhibits STAT5 in PTCL, leading to apoptotic cell death by means of the TRAIL/DR4 dependent extrinsic apoptotic pathway. Pimozide induced PTCL cell death is caspase 8 dependent, increases the expression of the TRAIL receptor, DR4, on the surface of pre-apoptotic PTCL cells, and enhances TRAIL induced apoptosis in a TRAIL dependent manner. In parallel, we show that mRNA and protein levels of intrinsic pathway BCL-2 family members and mitochondrial membrane potential remain unaffected by STAT5 knockdown and/or inhibition. In primary PTCL patient samples, pimozide inhibits STAT5 activation and induces apoptosis. Our data support a role for STAT5 inhibition in PTCL and implicate potential utility for inhibition of STAT5 and activation of the extrinsic apoptotic pathway as combination therapy in PTCL.

## INTRODUCTION

Peripheral T-cell lymphoma (PTCL) is an aggressive subset of Non-Hodgkin's lymphoma (NHL) with poor clinical outcomes, affecting ~7,000 individuals in the United States annually [1, 2]. The World Health Organization recognizes >25 subtypes of PTCL; each further characterized by heterogeneity including oncogenic mutations [1, 3, 4]. For the majority of these PTCL subtypes, patient mortality is not only high, but relapse is common and 5-year overall survival is 10-30% [2, 5, 6]. PTCL is currently treated with chemotherapy regimens designed for aggressive NHLs, but the rate and duration of response remain inferior to those of the B-cell lymphomas for which these treatments were designed [3, 7]. Recent clinical trials for treatment of PTCL include many novel agents, in addition to adaptations of traditional chemotherapy regimens. Novel therapeutic approaches under study include histone deacetylase (HDAC) inhibitors, immunoconjugates, antifolates, monoclonal antibodies, immunomodulatory agents, nucleoside analogues, proteasome inhibitors, and kinase inhibitors. Progress has been complicated by low numbers of PTCL patients and randomized prospective phase III data is currently limited, making it difficult to inform clinical management [5]. As such, there has been an increasing interest in identifying and developing additional therapeutic targets and treatment approaches to improve PTCL patient outcomes.

Research has been dedicated to the identification of potential oncogenic mutations and novel therapeutic targets in PTCL [8–19]. Several of these studies have revealed a recurrence of activating, oncogenic mutations in the JAK/STAT signaling pathway, specifically in the signal transducer and activator of transcription (STAT) STAT5B and STAT3 transcription factors, in multiple subtypes of PTCL [11, 15, 20–28]. The JAK/STAT signaling pathway plays an important role in T-cell activation during the immune response [29]. In physiologic JAK/STAT signaling through the common gamma chain, cytokine binding to the receptor complex triggers intracellular domain associated JAK1/3 molecules to autophosphorylate and activate the receptor, stimulating the recruitment and phosphorylation of STATs [30]. Activated STAT molecules dimerize and enter the nucleus to regulate the transcription of genes involved in cell proliferation and survival. Constitutive pathway activation, through mutation or overexpression of JAK1, JAK3, STAT3, STAT5, and the common gamma chain, have been reported in PTCL and shown to be oncogenic [11, 15, 31–34]. Of interest to our research, activating STAT5 mutations have been observed in multiple PTCL subtypes at frequencies of 21-36% in T-cell prolymphocytic leukemia (T-PLL), 33% in hepatosplenic T-cell lymphoma (HSTL), 63% in epitheliotropic intestinal T-cell lymphoma (EITL), 3% in Sézary syndrome (SS), and 2% in T-cell large granular lymphocytic leukemia (T-LGL) [11, 15, 20, 22–24]. The activating STAT5B mutation, N642H, which is observed repeatedly in PTCL, leads to a more aggressive clinical course in T-LGL and a significantly reduced probability of event free survival and an increase in cumulative incidence of relapse in T-cell acute lymphoblastic leukemia (T-ALL) [11, 35]. Activation of STAT3, both by mutation and overexpression, has also been observed in multiple PTCL subtypes [8, 10]. This is most prevalent in T-LGL, at a frequency of 40%, and also leads to poor patient outcomes [8, 10]. These studies demonstrate the substantial frequency of oncogenic STAT mutations in PTCL, particularly mutations in STAT5B.

Due to this prevalence of STAT5 mutations and their negative prognostic indications, inhibition of STAT molecules may be an appealing therapeutic approach in PTCL. JAK inhibitors have demonstrated clinical efficacy in hematologic malignancies driven by JAK activation, which has also been commonly reported in PTCL [36, 37]. However, the efficacy of JAK inhibition may be limited in cases with constitutively active STAT5, as STAT5 is downstream of these inhibitors' site of action. Thus, there is substantial interest in pursuing the development of downstream STAT5 inhibitors for the treatment of STAT5-driven PTCL. A high throughput screen identified the drug pimozide to specifically inhibit STAT5 in chronic myelogenous leukemia and acute myeloid leukemia with FLT3 mutations [38, 39]. Pimozide is FDA approved as a neuroleptic, although its exact mechanism of action remains unknown. We sought to gain a more complete understanding of the therapeutic potential of STAT5 inhibition in PTCL in order to help advance the development of STAT5 inhibitors for ultimate clinical application.

Our study furthers this goal by assessing the effect of STAT5 inhibition in PTCL. We evaluate the utility of STAT5 inhibition in PTCL cell lines as well as primary T-PLL patient samples and shed light on the mechanism by which pimozide induces PTCL cell death. This research aims to inform the development of STAT5 inhibitors as a novel therapeutic approach in this malignancy.

## RESULTS

### STAT5 is constitutively active in PTCL

**Figure 1:**
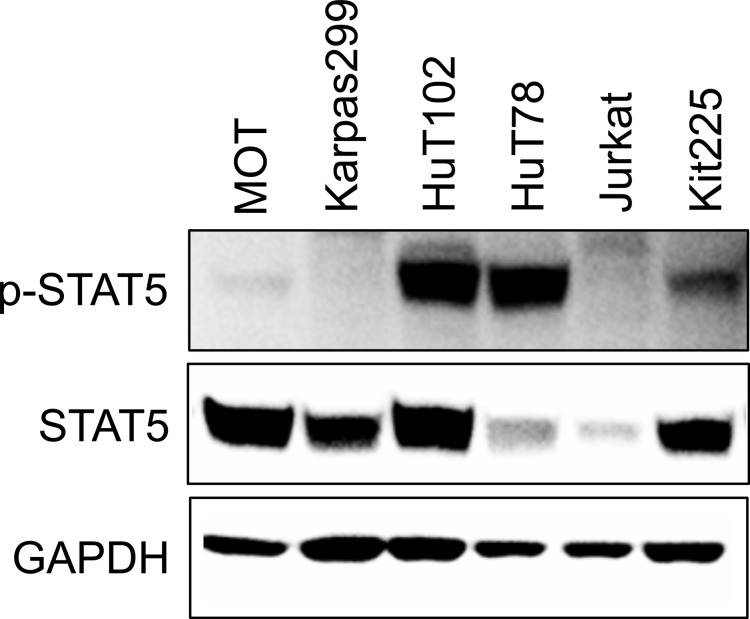
STAT5 is constitutively active in multiple PTCL cell lines Western blots show STAT5 and phospho-STAT5 expression in six T-cell malignancy derived cell lines in absence of cytokine stimulation.

To study the inhibition of STAT5 in PTCL, we identified patient derived cell line models. Analysis of six malignant T-cell lines cultured in the absence of cytokine stimulation revealed three lines, HuT102, HuT78, and Kit225, with high constitutive STAT5 activation (Figure 1). HuT102 and HuT78 were derived from PTCL patients with mycosis fungoides and SS subtypes, respectively, and are cytokine independent [40]. IL-2-dependent Kit225 cells were derived from a T-PLL patient, the same PTCL subtype as the patient samples included in this study [41]. We thus focused our analysis on Kit225 cells, as well as the HuT102 line based on its comparable level of STAT5 expression.

### Pimozide inhibits STAT5 in PTCL

**Figure 2:**
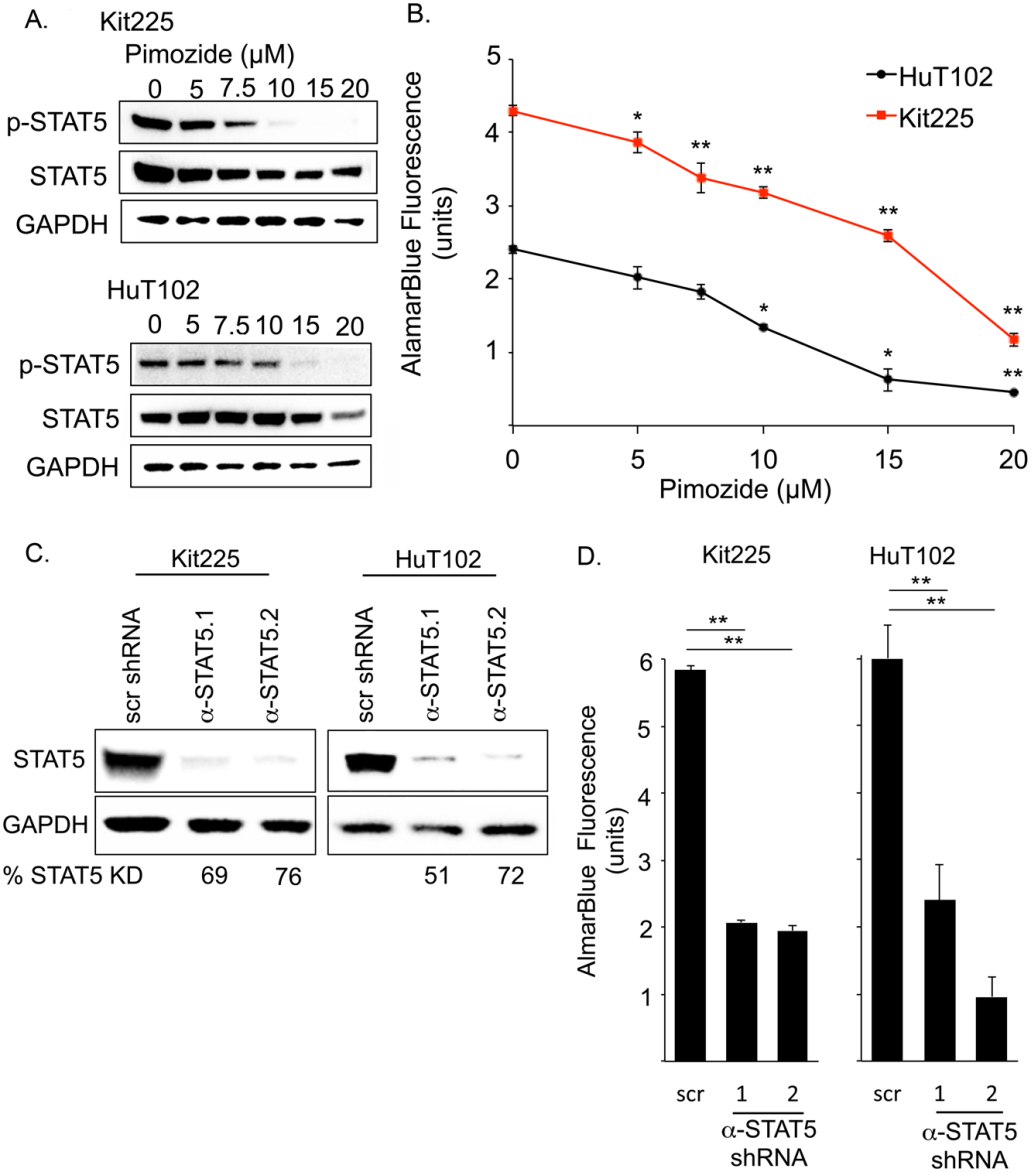
Disruption of STAT5 activity reduces PTCL cell viability **(A)** Western blots show phospho-STAT5 (Y694) (90kDa), STAT5 (90kDa), and GAPDH (37kDa) protein expression in PTCL cell lines. **(B)** Resazurin dye assay (AlamarBlue^©^) quantifies change in viable PTCL cell number after 48h with increasing pimozide concentrations. **(C)** Western blots show STAT5 expression 6 days after transduction with anti-STAT5 targeted shRNA (α-STAT5.1 and 2) in PTCL cell lines compared to non-targeted scramble (scr) control. **(D)** Graph of AlamarBlue^©^ assay quantifies PTCL cell viability. Significant p-values ^*^=*P*<0.05, ^**^=*P*<0.01.

To determine the ability of pimozide to inhibit STAT5 activation in PTCL, we cultured HuT102 and Kit225 cells in a range of drug concentrations for 48h [38, 39]. Immunoblots for p-STAT5 protein expression revealed a concentration dependent decrease in STAT5 phosphorylation (Figure 2A). We quantified the number of viable PTCL cells after culture with pimozide using resazurin dye assay and demonstrated a drug dose-dependent decrease in PTCL cell viability, which corresponded to the observed reduction in STAT5 activation for both cell lines (Figure 2A-2B). We calculated an IC_50_ for pimozide of 15μM in Kit225 and 11μM in HuT102 cells.

To explore whether this reduction in PTCL cell viability was due to STAT5 inhibition, we assessed the effect of STAT5 knockdown on PTCL cell viability. Two anti-STAT5 shRNA constructs reduced total STAT5 protein expression by >50% in all experiments (Figure 2C). Both PTCL cell lines demonstrated an approximately 2/3 reduction in viable cell number after STAT5 knockdown, as compared to each cell line transduced with control shRNA (Figure 2D). These experiments show that reduction of STAT5 expression reduces PTCL cell viability.

### Pimozide induces apoptosis in PTCL cells

**Figure 3:**
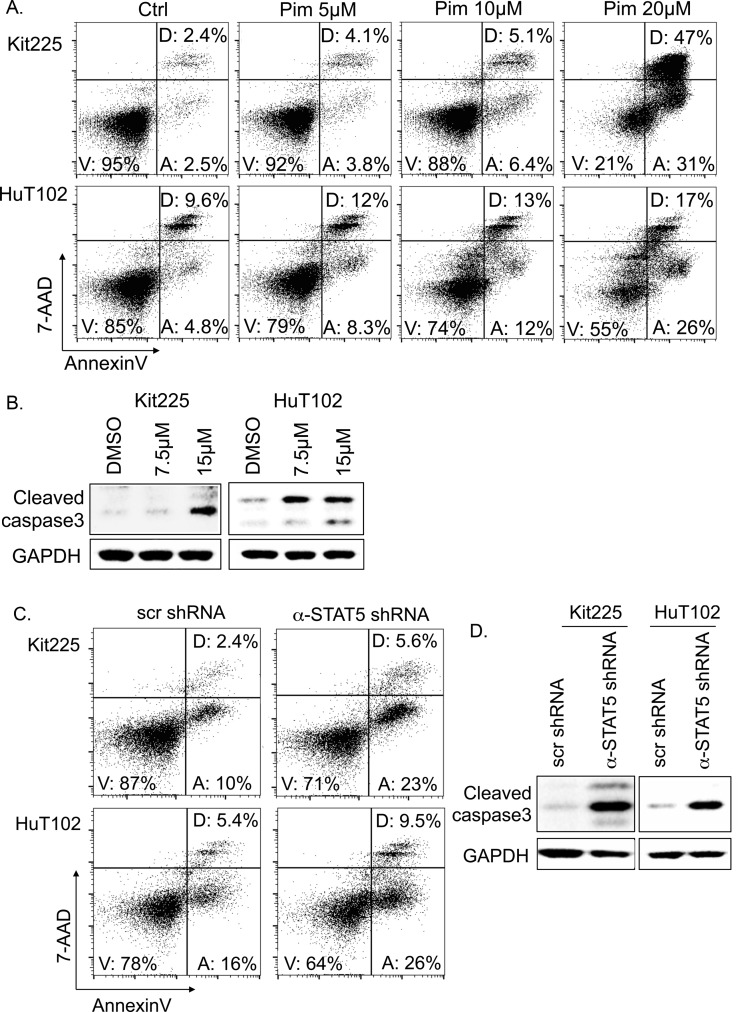
Apoptosis in PTCL cells following STAT5 knockdown and inhibition **(A)** Flow cytometry identifies percentage of early apoptotic (“A”), viable (“V”), and dead/late apoptotic cells (“D”) by APC-AnnexinV/7-AAD following 48h with indicated pimozide concentrations. At least 40,000 events were collected. **(B)** Western blots show protein expression of cleaved caspase 3 (17 and 19kDa) versus GAPDH control (37 kDa). **(C)** Flow cytometry indicates proportion of apoptotic cells 6 days after anti-STAT5 shRNA knockdown verses control (scr). **(D)** Western blots show protein expression of cleaved caspase 3 versus GAPDH.

We demonstrated that PTCL cell viability is diminished in response to inhibition of STAT5 phosphorylation by pharmacologic and genetic means. To determine the mechanism by which pimozide reduces viable PTCL cell number, we analyzed PTCL cell death for evidence of apoptosis over a range of pimozide concentrations. PTCL cells were cultured with pimozide for 48h and assessed by flow cytometry. Evidence of early apoptotic cell death includes positive stain by AnnexinV, while remaining 7-AAD negative. Double positive cells are indicative of late-stage apoptosis or another cell death mechanism [42]. Our results demonstrated pimozide induced a concentration dependent increase in the proportion of PTCL cells undergoing apoptosis (Figure 3A). Apoptotic Kit225 cells increased from 2.5% to >30% with 20μM of pimozide compared to control. In Hut102 cells, apoptosis increased ~5 fold, from <5% to >25% of cells. Cell death due to apoptosis for both cell lines was furthermore confirmed by immunoblot for caspase 3 cleavage (Figure 3B).

To investigate if induction of apoptosis is related to pimozide's STAT5 inhibition, we also assessed PTCL cell survival after STAT5 knockdown. In Kit225 cells expressing <30% of endogenous STAT5 levels, apoptotic cells were increased from 10% to 23% verses control (Figure 3C). Apoptotic HuT102 cells increased from 16% to 26% after STAT5 knockdown. Apoptosis was confirmed as the mechanism of cell death in both cell lines using immunoblot for cleaved caspase 3 activation (Figure 3D).

### STAT5 knockdown does not decrease BCL-2 family member expression in PTCL cell lines

**Figure 4:**
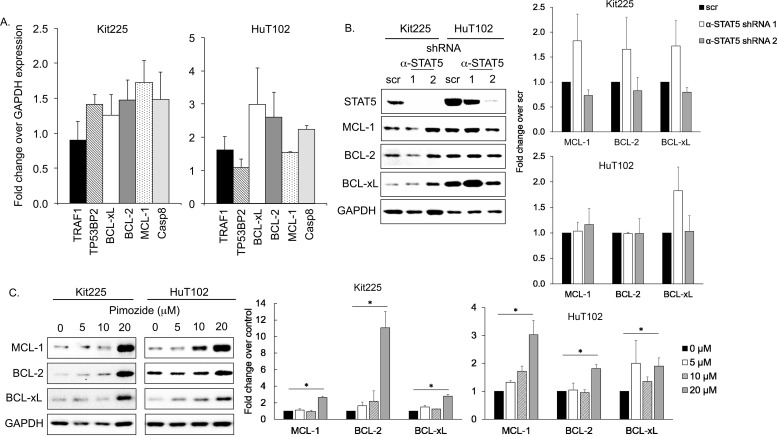
Expression of anti-apoptotic BCL-2 family after STAT5 knockdown/inhibition **(A)** mRNA expression of six apoptotic mediators regulated by STAT5 relative to GAPDH control by RT qPCR in PTCL cell lines after STAT5 knockdown. **(B)** Western blots show protein expression of three anti-apoptotic BCL-2 family members after STAT5 knockdown with shRNA (α-STAT5.1 and 2) versus control (scr). Quantification bar graphs are shown to the right. Variations are not statistically significant. **(C)** MCL-1 (40 kDa), BCL-2 (28 kDa), BCL-xL (30 kDa), and GAPDH protein expression after 48h in indicated concentration of pimozide. Quantification bar graphs are shown to the right, ^*^=P<0.05.

To evaluate the mechanism by which STAT5 knockdown and inhibition with pimozide induced apoptosis in PTCL, we next studied molecules influencing apoptosis that are subject to transcriptional regulation by STAT5 in human T-cells [43–47]. Apoptotic regulators regulated by STAT5 in human CD4+ T-cells included six candidate molecules: MCL-1, BCL-2, BCL-xL, TRAF1, TP53BP2, and caspase 8 (Casp8), [47, 48]. The mRNA expression of these candidate molecules was assessed in PTCL cell lines after STAT5 knockdown and no significant differences in expression were observed compared to control conditions (Figure 4A). Similarly, protein levels of anti-apoptotic BCL-2 family members BCL-xL, BCL-2, and MCL-1 were not significantly altered, despite indication that STAT5 regulates apoptosis via their transcriptional downregulation in other models (Figure 4B) [45, 47, 49–51].

We also assessed BCL-2, BCL-xL, and MCL-1 protein expression in HuT102 and Kit225 after culture with pimozide. Our data demonstrated no significant change in expression by immunoblot when cultured with 5-10μM of pimozide for 48h (Figure 4C). Protein expression of these anti-apoptotic molecules were increased following 20μM pimozide, which may be compensatory or due to general organelle decomposition. Our results do not support a role for BCL-2 family members contributing to apoptosis following STAT5 inhibition in our PTCL models.

We next sought to determine if increased apoptosis was due to a collapse of metabolic mitochondrial function by assessing the bioenergetic profile of PTCL cell lines through ATP production in the context of STAT5 inhibition. ATP bioluminescent assay demonstrated that, in HuT102 and Kit225 cell lines, ATP production decreases in a pimozide dependent manner (Supplementary Figure 1). ATP production was also significantly reduced in both cell lines following STAT5 knockdown (Supplementary Figure 2). These data suggest that inhibition of STAT5 interferes with metabolic function of PTCL cell lines. Based on these results, pimozide likely induces apoptosis in PTCL cells by means of interference with cellular metabolism outside of the context of BCL-2 family survival protein expression.

### Pimozide induces apoptosis via the TRAIL/DR4 dependent extrinsic apoptotic pathway

**Figure 5:**
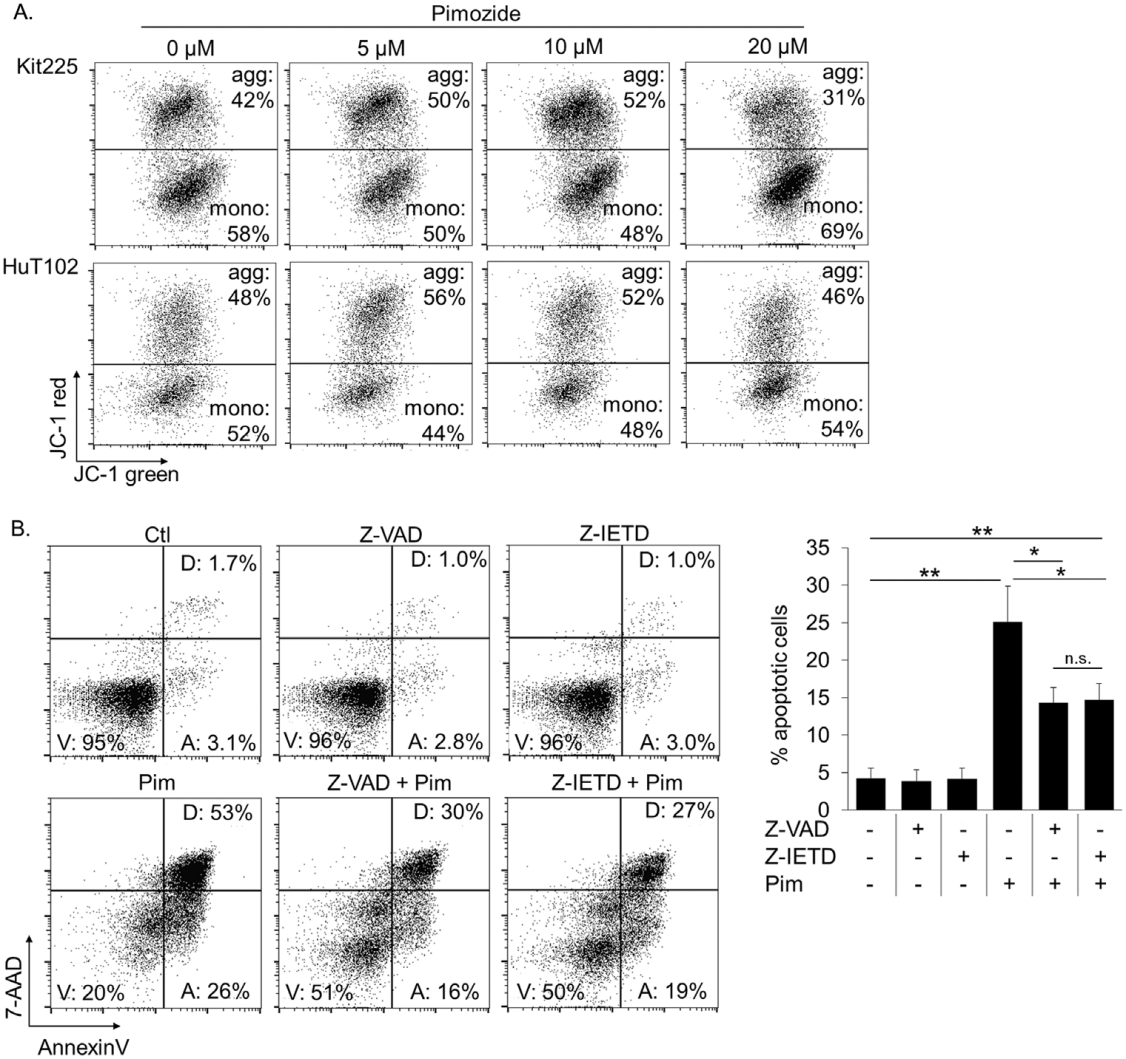
Pimozide induced apoptosis is caspase 8 dependent **(A)** AnnenxinV negative pre-gated FACS plots show proportion of mitochondria with viable (JC-1 hi, aggregates) versus compromised (JC-1 red low, monomers) membrane integrity following 24h pimozide. **(B)** Proportion of PTCL cells undergoing apoptosis following 24h pimozide 20μM +/− pretreatment with caspase 8 inhibitor, Z-IETD, or pan-caspase inhibitor, Z-VAD. Gates “V,” “A,” and “D” contain viable, early apoptotic, and late apoptotic/dead cells, respectively. Significance indicated ^*^=*P*<0.005, ^**^=*P*<0.0005.

We subsequently explored whether the mechanism of apoptosis involves the intrinsic/mitochondrial pathway or extrinsic/death receptor pathway. First, we assessed changes in mitochondrial membrane potential (MMP) in apoptotic PTCL cells by JC-1 dye [52]. Accounting for slight decrease in MMP with advanced stages of apoptosis, our results indicate no significant change in MMP in pre-apoptotic PTCL cells cultured with pimozide versus control (Figure 5A). These results do not support the intrinsic pathway of apoptosis as a mechanism of cell death in PTCL cells treated with pimozide.

We next investigated the extrinsic pathway as a potential mechanism for pimozide induced apoptosis. The extrinsic apoptotic pathway signals through death receptors to activate caspases, bypassing the mitochondria [53–55]. Caspase 8 activation is exclusive to the extrinsic pathway. We compared the abilities of a caspase 8 inhibitor, Z-IETD-FMK, and a pan-caspase inhibitor, Z-VAD-FMK, which prevents apoptosis through both extrinsic and intrinsic pathways, to prevent apoptosis in pimozide treated PTCL cells. Significantly fewer pimozide treated cells underwent apoptosis after pretreatment with either of the caspase inhibitors, indicating that apoptosis is occurring by means of caspase 8 activity in this context (Figure 5B). Thus, our data suggest that pimozide induces cell death in PTCL cells by means of the extrinsic apoptotic pathway.

**Figure 6:**
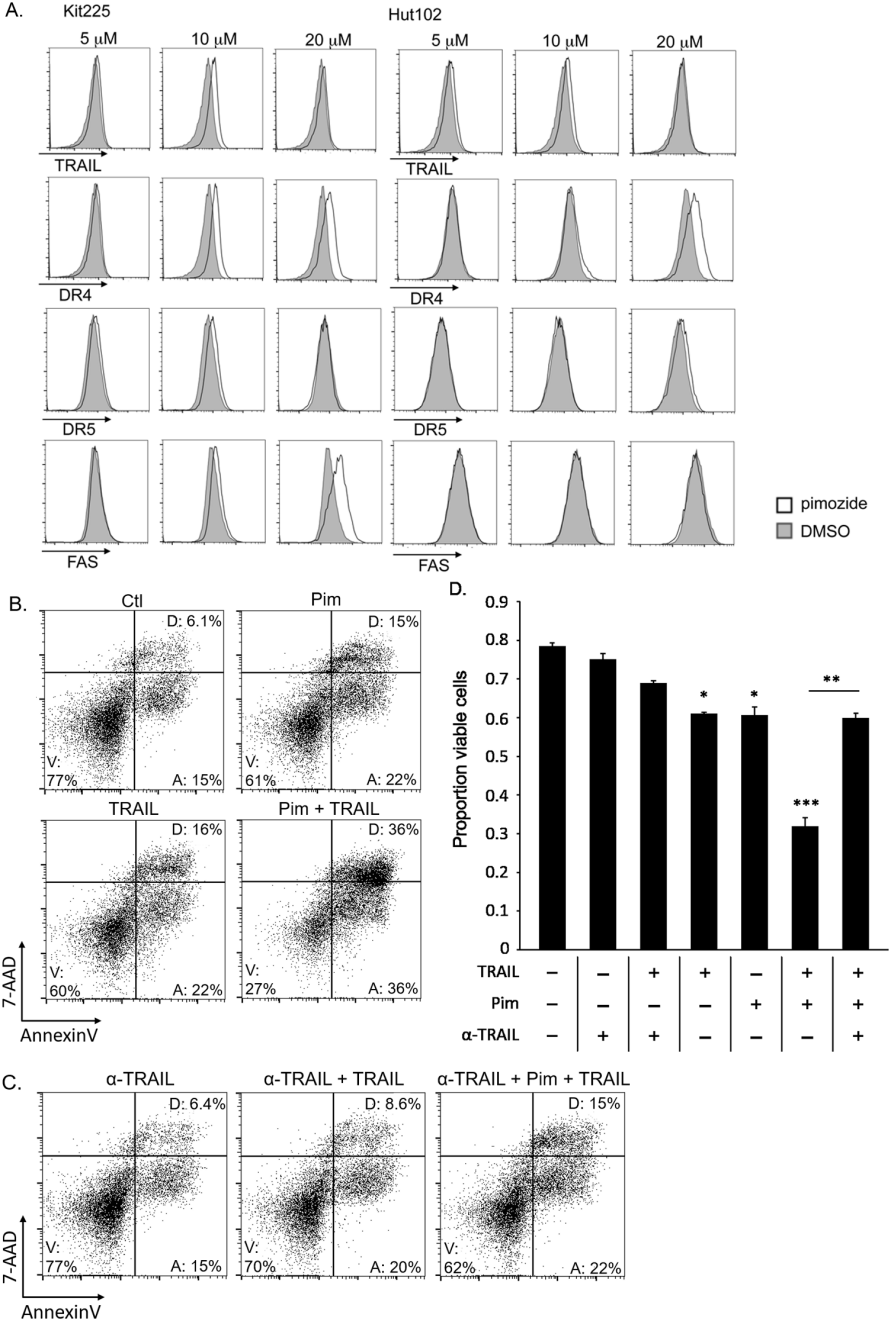
Pimozide enhances TRAIL/DR4 dependent apoptosis in PTCL **(A)** Histograms show difference in TRAIL, DR4, DR5, and FAS surface expression on AnnexinV negative Kit225 and HuT102 cells after 48h pimozide (white) versus control (gray). **(B)** FACS plots show viable Kit225 cells with combination of 15μM pimozide and 10 ng/mL TRAIL after 24h. **(C)** FACS plots show viable cells from same experiment shown above with addition of TRAIL neutralizing antibody (α-TRAIL). **(D)** Bar graph quantifies viable (AnnexinV, 7-AAD negative) PTCL cells from 3 independent experiments shown in parts B and C. The 4^th^, 5^th^, and 6^th^ bars are significant compared to the first three control bars at P value indicated, ^*^=P<0.05, ^**^=P<0.01, ^***^=P<0.005.

The extrinsic pathway is initiated by ligand (FasL, TNF-α, TRAIL) binding to corresponding death receptors (FAS, TNFαR, DR4/DR5) on the cell surface [55]. To investigate this pathway's involvement further, we assessed several cell surface death receptors after culture with pimozide for changes in expression. We observed that expression of the TRAIL death receptor DR4 (TRAIL-R1) was upregulated in a pimozide concentration-dependent manner (Figure 6A). FAS expression was also upregulated on the surface of Kit225 cells. Combination of pimozide and TRAIL enhanced Kit225 cell death *in vitro* (Figure 6B). Addition of a TRAIL neutralizing antibody restored cells to near baseline levels of apoptosis, supporting that this cell death is TRAIL dependent (Figure 6C). These results suggest that TRAIL/DR4 signaling may be involved in the mechanism of pimozide induced apoptosis in PTCL cells.

### Pimozide inhibits STAT5 and induces apoptosis in primary patient PTCL

**Figure 7:**
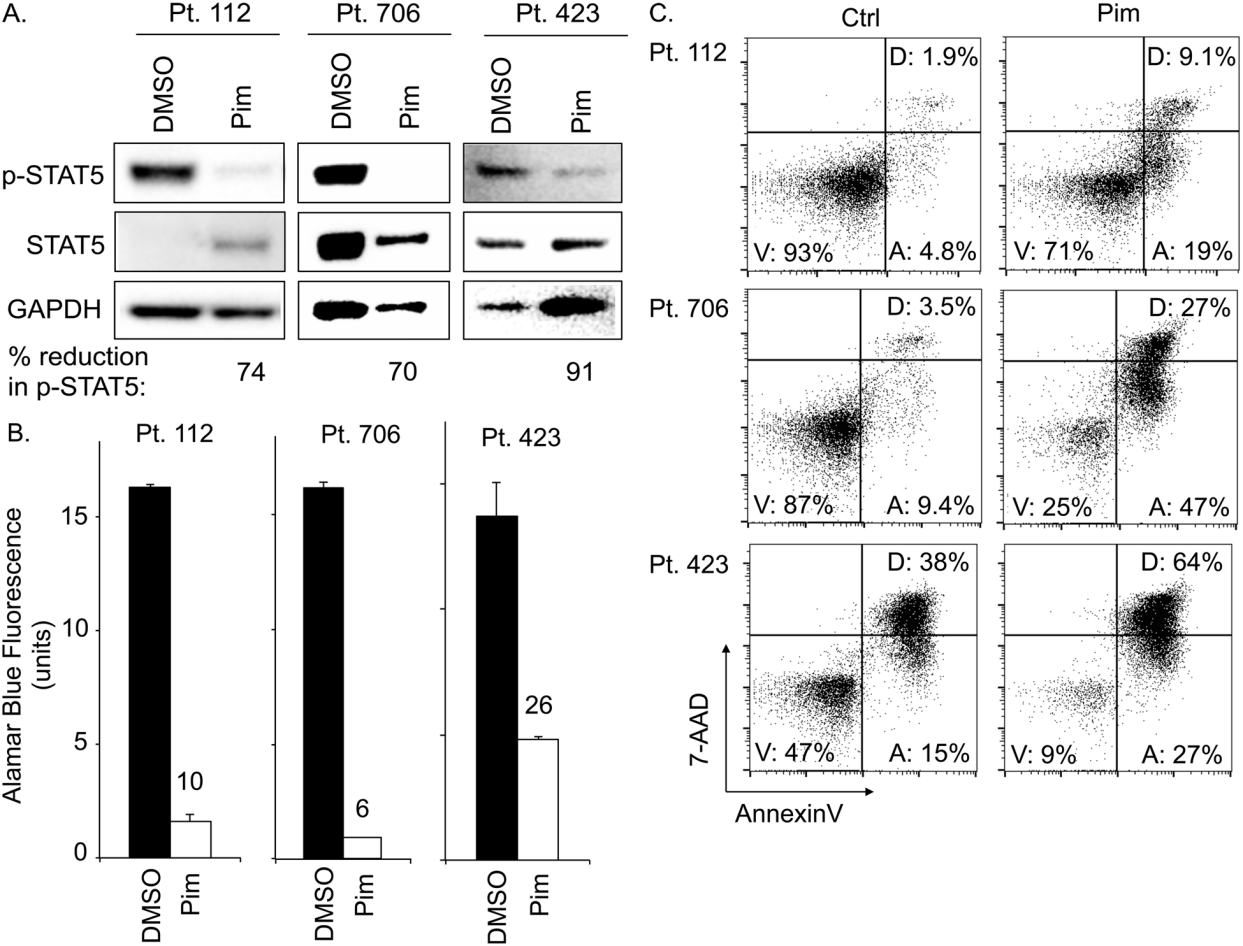
Pimozide inhibits STAT5 activation and induces apoptosis in primary PTCL patient samples **(A)** Protein expression of phospho-STAT5, STAT5, and GAPDH for three different *ex vivo* PTCL patient samples (T-PLL subtype) after 24h pimozide 20μM versus control (Ctrl). **(B)** AlamarBlue^©^ assay quantifies viable cells from PTCL patient samples after 48h pimozide versus control. **(C)** FACS plots show proportion of apoptotic patient PTCL cells (“A”) after 48h *ex vivo* culture with 20μM pimozide versus control.

To assess our findings in patient primary malignant PTCL cells, we investigated the effect of pimozide on T-PLL patient samples *ex vivo*. Patient samples maintained under dimethyl sulfoxide (DMSO) vehicle conditions had substantial STAT5 phosphorylation, supporting the prevalence of STAT5 activation in T-PLL (Figure 7A). In comparison, pimozide reduced p-STAT5 expression by ≥70% in all samples. These results support that pimozide inhibits STAT5 in primary PTCL, consistent with the findings from established cell lines. Pimozide also reduced the number of viable primary PTCL cells, resulting in a 74-94% decrease in viability verses control, due to increased apoptotic cell death (Figure 7B-7C). Percent of apoptotic primary PTCL cells increased in all samples by an average of ~20% versus control. Our data, therefore, support pimozide induced STAT5 inhibition in primary patient PTCL.

## DISCUSSION

We explore STAT5 as a therapeutic target in PTCL. Activating STAT5 mutations have been observed in multiple PTCL subtypes and are associated with a more aggressive clinical course [11, 15, 20, 22–25, 35]. In hematologic malignancies with activating JAK mutations, JAK inhibitors have proved clinically useful, however, they target upstream of STAT5 and may be ineffective in PTCL driven by activating STAT5 mutations [15, 36, 37]. Thus, STAT5 inhibition is a promising approach. We show that p-STAT5 is important in propagation of PTCL, as studied in two cell lines and in three patient samples. When inhibited by pharmacologic or genetic means, PTCL cell viability is reduced through induction of TRAIL mediated apoptosis. These results demonstrate that pimozide inhibits STAT5 and support the utility of STAT5 inhibition as a therapeutic strategy in PTCL.

We provide initial evidence of a mechanism by which STAT5 inhibition with pimozide induces apoptosis. Previous research demonstrates that pimozide decreases viability of two T-cell lines and two T-PLL patient cases *in vitro* [15], and the work presented here extends those findings to include a mechanism for evidence of cell death. We show that pimozide reduces PTCL cell viability in two additional cell lines and three T-PLL patient samples and this induction of apoptosis is caspase 8 and TRAIL dependent, associated with upregulation of the cell surface expression of TRAIL death receptor, DR4. These results support that pimozide induces apoptosis in PTCL cells via the extrinsic, TRAIL/DR4 dependent, apoptotic pathway. A study by Kanai, *et. al.* utilized chromatin immunoprecipitation with sequencing (ChIP-seq) with qPCR validation to identify STAT5A and STAT5B targeted genes in human CD4+ T-cells following 3 days in culture with IL-2 [47]. Their data show that TRAIL, also known as TNFSF10, is dominantly regulated by STAT5B. STAT5B was found to bind directly to the regulatory sequence “TTCCAAGAA” in the TRAIL promoter. These findings, in conjunction with our own, support that TRAIL induced cell death may be regulated by STAT5 and suggest a mechanism for apoptosis induced by STAT5 inhibition. In context, our results provide insight into targeting PTCL cells and improve our understanding of an incompletely characterized pharmaceutical for STAT5 inhibition.

It is noteworthy that BCL-2, BCL-xL, and MCL-1 do not appear to play a role in the induction of apoptosis following STAT5 knockdown or inhibition in our analysis. Prior research by others suggests that STAT5 knockdown triggers apoptosis through anti-apoptotic BCL-2 signaling via the intrinsic pathway in various hematologic malignancies and non-malignant T-cells [45, 47, 49–51]. However, in our study, BCL-2, BCL-xL, and MCL-1 expression were not decreased after STAT5 knockdown. MMP was also not affected by STAT5 inhibition, suggesting that pimozide does not induce apoptosis via the intrinsic, BCL-2 family dependent, pathway. This finding may represent a mechanism unique to pimozide and/or PTCL.

Targeting the JAK/STAT and apoptotic pathways in combination presents a promising therapeutic approach in PTCL. Combination therapies with BCL-2/BCL-xL inhibitors, targeting the intrinsic pathway, and JAK inhibitor ruxolitinib have demonstrated synergy in pre-clinical models of adult T-cell leukemia lymphoma and T-ALL [25, 56, 57]. Our research suggests that targeting the extrinsic pathway may offer a mechanistic advantage and implicates a role for combination of STAT5 inhibition with death receptor signaling agonists as a future therapeutic approach in PTCL, although effective TRAIL agonists are still in development [58].

It is important to note, that while inhibition of STAT5 is promising for PTCL therapy, alternative STAT5 inhibitors, aside from pimozide, may have greater clinical potential. Pimozide is FDA approved and has been used for decades as a neuroleptic with manageable side effects. Clinical dosing, however, is recommended not to exceed 10 mg/day and it is unknown if pimozide has any effect as a STAT5 inhibitor *in vivo*. Novel applications of pimozide are currently being studied in just over a dozen clinical trials for psychotic and neurologic disorders including schizophrenia, ALS, and tic disorders (clinicaltrials.gov). Furthermore, pimozide has not been shown to be a direct STAT5 inhibitor [38, 39]. Other STAT5 inhibitors have been developed specifically for direct, targeted STAT5 inhibition. Promising among these is a compound referred to as 13a, which was developed using *in silico* modeling and demonstrated to specifically inhibit STAT5 with an IC_50_ of ~3.5μM in a FLT3-ICD driven cell line [59]. Application of this and other novel specific targeted STAT5 inhibitors in primary patient PTCL samples will be an important future direction of this research before clinical utility can be assessed.

Although substantial pre-clinical research remains to be conducted with STAT5 inhibitors prior to their application in clinical trials, clinical trials with many other agents are ongoing for PTCL. As a result of recent trials, two HDAC inhibitors, romidepsin and belinostat, have been approved by the FDA for use in relapsed/refractory PTCL [3, 60, 61]. Immunoconjugates, such as brentuximab vedotin, have been studied in clinical trials in PTCL patients with promising results [62–65]. Several monoclonal antibodies are also in development. Alemtuzumab (anti-CD52), mogamulizumab (anti-CCR4), and zanolimumab (anti-CD4) have all been shown to induce a complete remission in a fraction of patients with relapse/refractory PTCL [3, 66, 67]. Studies of other agents such as lenalidomide, nucleoside analogs, proteasome inhibitors, the mTOR inhibitor everolimus, and the JAK inhibitor ruxolitinib are also currently in clinical trials for PTCL [1–3, 68–70]. These agents may demonstrate clinical utility alone, or in combination with current therapeutic options.

In addition to activating mutations in STAT5, JAK mutations have also been identified in PTCL. Constitutively activating mutations in JAK kinases, JAK1 and JAK3, have been observed in approximately one third to one half of cases of the T-PLL subtype of PTCL [15, 24, 34, 71, 72]. Furthermore, T-PLL patients with activating JAK3 mutations have experienced inferior clinical outcomes, including significantly decreased overall survival [15]. This observation supports application of JAK-targeted tyrosine kinase inhibitors in PTCL. JAK inhibitors ruxolitinib and tofacitinib have been shown to limit the proliferation of several different malignant T-cell lines as well as primary patient cells expressing activated JAK mutations *ex vivo* [25, 32, 73–76]. It is important to note, however, that JAK inhibitor efficacy is dependent on the mechanism of JAK/STAT pathway activation. In PTCL cases where STATs are directly activated without involving upstream signaling, JAK inhibitors have proven ineffective [15, 21, 77, 78]. Combination of JAK and STAT5 inhibition may increase efficacy and minimize development of drug resistance. Our future research will explore some of these applications.

In conclusion, this study determines that targeting STAT5 is an effective therapeutic approach in PTCL and has the potential to be further developed for clinical application. We show that inhibition of STAT5 induces apoptosis in primary PTCL cells and are the first to demonstrate that STAT5 inhibition with pimozide induces apoptosis via the TRAIL/DR4-dependent extrinsic pathway. Our novel understanding of this mechanism may provide opportunities for new clinical and research applications. Our data suggest that PTCL patients with activating STAT5 mutations could benefit from STAT5 inhibitors either as mono-therapy or in combination with additional therapeutic strategies to provide more efficacious, less toxic treatment.

## MATERIALS AND METHODS

### Primary PTCL specimens

Peripheral blood specimens were collected from PTCL patients at the University of Maryland Greenebaum Comprehensive Cancer Center with the approval of the University of Maryland, Baltimore Institutional Review Board (UMB IRB). Written consent was obtained from all patients using a UMB IRB approved consent procedure. Peripheral blood mononuclear cells were isolated from each specimen by Ficoll gradient centrifugation according to the manufacturer's protocol (GE Healthcare). Cells were cultured for up to 48h with IL-2 stimulation.

### Flow cytometry

We performed flow cytometry on PTCL cell lines, HuT102 and Kit225, as well as primary PTCL samples. Cells were stained with AnnexinV-APC (eBioscience, Santa Clara, CA) and 7-AAD (7-amino actinomycin D) viability solution (BioLegend, San Diego, CA) to assay for apoptosis. MMP was assayed by staining with JC-1 dye (eBioscience) and AnnexinV. Cell surface expression of death receptors was determined by flow cytometry with separate stains using PE-conjugated anti-human CD253 (TRAIL; clone: RIK-2), PE-conjugated anti-human CD261 (DR4, TRAIL-R1; clone: DJR1), PE-conjugated anti-human CD262 (DR5, TRAIL-R2; clone: DJR2-4), FITC-conjugated anti-human CD95 (FAS; clone: DX2) (BioLegend).

### Cell culture and conditions

Malignant human T-cell lines studied include Jurkat, Karpas299, HuT78, MoT, and HuT102, supplied by ATCC, and Kit225 provided by Dr. Thomas Waldmann at the National Institutes of Health. All lines were cultured in RPMI1640 medium with 10% FBS and maintained at a density below 1×10^6^ cells/mL. Kit225, an IL-2 dependent cell line, was cultured with 100U/mL of IL-2. Primary patient PTCL cells were cultured in RPMI with 10% FBS and 100U/mL of IL-2 for *ex vivo* experiments. PTCL cells were treated with pimozide (Sigma Aldrich, St. Louis, MO) at a range of concentrations from 5-20μM for 24-48h with DMSO vehicle not exceeding 0.01% in culture. Kit225 cells were cultured with recombinant human TRAIL at 1-10 ng/mL +/− pimozide for 24h (Biolegend). TRAIL neutralization was performed with human anti-TRAIL antibody at 10μg/ml for 24h (clone # 75411, R&D Systems).

### Anti-STAT5 shRNA knockdown

Four different anti-STAT5A and four different anti-STAT5B human lentiviral shRNA sequences in puromycin selective pLKO.1 plasmids were obtained from The RNAi Consortium (GE Dharmacon; clone IDs TRCN0000019304-8, TRCN0000019354-8). A control scramble shRNA, not targeted to the human genome, also in pLKO.1 plasmid was provided by Dr. Tami Kingsbury at the University of Maryland, Baltimore. Anti-STAT5 lentivirus for transduction was generated by calcium phosphate transfection of HEK293T cells as previously described using 7.5μg of shRNA plasmid, 5μg of pSP2, and 2.5μg of pMD2G viral helper plasmids [79]. PTCL cell lines were transduced with each anti-STAT5 shRNA plasmid using RetroNectin^®^ according to the manufacturer's Supernatant Infection Method protocol (Takara, Japan). Clones TRCN0000019354 and TRCN0000019357, referred to as anti-STAT5 shRNA 1 and 2, respectively in this study, consistently produced the most efficient knockdown of total STAT5 expression and were selected for subsequent experiments.

### Western blot analysis

Cells were lysed with NP-40 lysis buffer and protein concentration was determined by Bradford assay. Samples were subjected to gel electrophoresis and transferred to PVDF membrane by dry transfer (iBlot; Invitrogen). Membranes were blocked for 1h with 5% BSA. Primary antibodies for p-STAT5 (Y694), GAPDH (14C10), BCL-xL, BCL-2 (D55G8), MCL-1 (D5V5L) and cleaved caspase 3 (D175) (Cell Signaling Technology) and STAT5 (sc-835, Santa Cruz Biotechnology) were used. Primary antibodies were diluted 1:1000 in 1% BSA and incubated overnight at 4°C. Anti-rabbit IgG HRP secondary antibody (Cell Signaling Technology) diluted 1:2000 was incubated for 1h at room temperature. Blots were developed using ECL Prime Western Blotting Detection Reagent (GE Healthcare) and imaged/analyzed using Image Lab software (BioRad).

### Cell viability assays

The number of viable cells was quantified with trypan blue viability dye by automated cell counter (Countess, Invitrogen). Viability was also assessed by resazurin dye (AlamarBlue^©^) assay according to the manufacturer's protocol (Invitrogen), imaged by spectrophotometer (Perkin Elmer Victor X3 workstation).

### Apoptosis assays

Proportions of PTCL cells undergoing apoptosis were assessed by flow cytometry following staining with AnnexinV (eBioscience) and 7-AAD (BioLegend) according to the manufacturer's protocols. Apoptosis was also determined by Western blot for caspase 3 cleavage. We assessed involvement of the extrinsic pathway using caspase inhibitors. Cells were pretreated with 50μM of a caspase 8 inhibitor, Z-IETD-FMK, or a pan-caspase inhibitor, Z-VAD-FMK, for 30min, then cultured with 5-20μM pimozide or DMSO for 24h (BD Biosciences). Involvement of the intrinsic pathway was assessed using JC-1 MMP dye according to the manufacturer's protocol (eBioscience).

### ATP production assay

Bioenergetic profile of PTCL cell lines cultured with varying concentrations of pimozide (5-20μM) and following anti-STAT5 shRNA knockdown vs. control was assessed using a ATP bioluminescent somatic cell assay kit according to the manufacturer's protocol (SigmaAldrich). Imaging was performed by spectrophotometer (Perkin Elmer Victor X3 workstation).

### RNA isolation and quantitative real-time RT-PCR

RNA was isolated using RNAqueous kit according to the manufacturer's protocol (Life Technologies). Reverse transcription was performed according to the First-Strand cDNA Synthesis protocol (Thermo Fischer Scientific, Halethorpe, MD). Real-time quantitative PCR (qPCR) was performed using Taqman probes for human TRAF1, TP53BP2, Casp8, BCL-2, BCL-xL, and MCL-1 (Thermo Fischer Scientific) using the QuantStudio 6 Flex (Applied Biosystems Life Technologies) according to the manufacturer's protocol.

### Statistics

All data are representative of at least 3 independent experiments. Results were expressed as mean ± standard error of the mean. ANOVA and two-tailed student T-test were used to determine significance. P-values less than 0.05 were considered significant.

## SUPPLEMENTARY MATERIALS FIGURES AND TABLES



## Abbreviations

ATP: Adenosine triphosphate
Casp8: Caspase 8
Ctrl: Control
DMSO: Dimethyl sulfoxide
EITL: Epitheliotropic intestinal T-cell lymphoma
HSTL: Hepatosplenic T-cell lymphoma
MMP: Mitochondrial membrane potential
NHL: Non-Hodgkin's lymphoma
PTCL: Peripheral T-cell lymphoma
Scr: Scramble
SS: Sézary syndrome
STAT: Signal transducer and activator of transcription
T-ALL: T-cell acute lymphoblastic leukemia
T-LGL: T-cell large granular lymphocytic leukemia
T-PLL: T-cell prolymphocytic leukemia
TRAIL: TNF-related apoptosis-inducing ligand
UMB IRB: University of Maryland, Baltimore Institutional Review Board
