# Supplementary material for: STAT5 inhibition induces TRAIL/DR4 dependent apoptosis in peripheral T-cell lymphoma
Source: Oncotarget. 2018 Mar 30;9(24):16792–806. doi: 10.18632/oncotarget.24698 (PMC5908286; doi:10.18632/oncotarget.24698)
Supplement: Supplementary file 1 [file oncotarget-09-16792-s001.pdf]

STAT5 inhibition induces TRAIL/DR4 dependent apoptosis in peripheral T-cell lymphoma

SUPPLEMENTARY MATERIALS

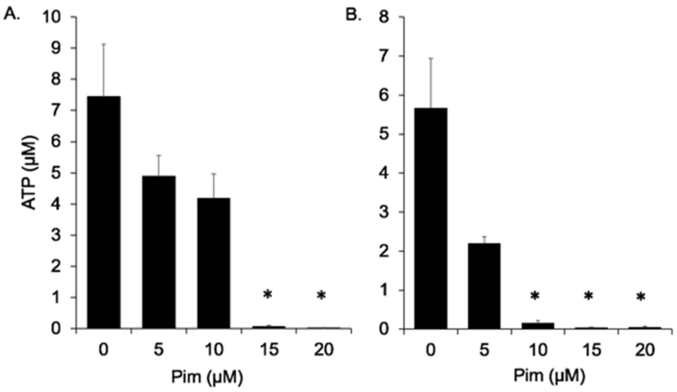

Supplementary Figure 1: Pimozide significantly decreases ATP production in PTCL cell lines. ATP concentration in **(A)** Kit225 and **(B)** HuT102 cells following 48h culture with indicated concentrations of pimozide is shown, calculated by ATP bioluminescence assay, *= $P<0.05$.

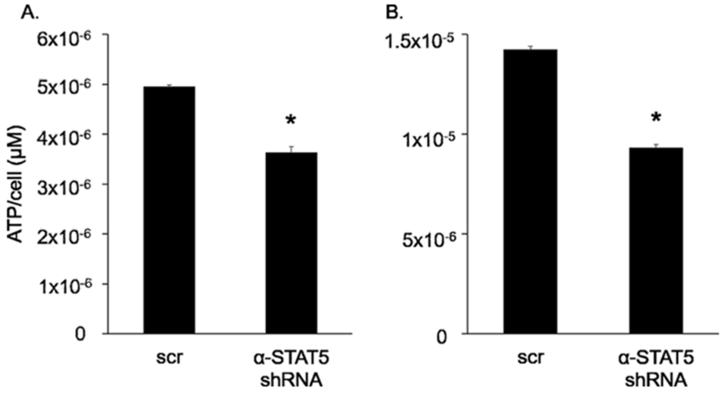

Supplementary Figure 2: Knockdown of STAT5 significantly decreases ATP production in PTCL cell lines. ATP concentration in **(A)** Kit225 and **(B)** HuT102 cells is shown 7 days after transduction with anti-STAT5 targeted shRNA, calculated by ATP bioluminescence assay. Transduced cells were selected in puromycin 2μg/ml for 3 days. ATP concentration is reported per viable cell to account for variations in transduction efficiency, *= $P<0.05$.
